# Reconstruction of a fibrous pseudo-patella in total knee arthroplasty after patellectomy: a function-preserving strategy via intraoperative soft tissue balancing and postoperative rehabilitation– a case report

**DOI:** 10.3389/fsurg.2026.1953154

**Published:** 2026-10-23

**Authors:** Zhibing Wang, Xingwang Cheng, Xia Zhang

**Affiliations:** 1 Sichuan Mianyang 404 Hospital, Mianyang, Sichuan, China; 2 The Second Affiliated Hospital of Chongqing Medical University, Chongqing, China; 3 Army Medical University Xinqiao Hospital Department of Orthopeadics, Chongqing, China

**Keywords:** fibrous pseudo-patella, patellectomy, postoperative rehabilitation, post-patellectomy osteoarthritis, quadriceps strength, soft tissue balancing, total knee arthroplasty

## Abstract

**Background:**

To report the clinical outcomes in a case of severe osteoarthritis secondary to patellectomy 40 years earlier and successfully treated with the function-preserving strategy via fibrous scar tissue extension in total knee arthroplasty (TKA) and postoperative rehabilitation program.

**Methods:**

A 59-year-old male patient presented with severe osteoarthritis secondary to the left patellectomy due to trauma 40 years earlier. Intraoperative exploration revealed dense fibrous scar tissues measuring approximately 4.0 cm × 3.0 cm × 1.5 cm at the original patellectomy site. The patient was treated with a function-preserving strategy involving preservation and reshaping of residual fibrous scar tissue into a fibrous pseudo-patella prior to TKA and postoperative rehabilitation, instead of traditional bony patella reconstruction. The fibrous scar tissues acted as a natural alternative to the patella (fibrous pseudo-patella) through extensive lateral retinaculum release and medial capsular tightening to balance soft tissues, and allowed the further TKA using a semi-constrained prosthesis. Postoperatively, the patient was managed by a rehabilitation program for strengthening the quadriceps femoris muscle. Follow-up visits were conducted at 6 weeks, 3 months, and 1 year.

**Results:**

The patient benefited from an immediate postoperative pain relief of the knee. Quadriceps strength increased markedly at 6 weeks postoperatively, indicating a full restoration of active knee extension. At the 3-month follow-up, the patient regained functional knee range of motion (ROM) of 0°−120°, and was able to independently squat and stand. The 1-year imaging assessment showed an optimal prosthesis positioning and a stable trajectory of the fibrous pseudo-patella. In addition, the patient presented normal gait patterns at 1 year postoperatively.

**Conclusion:**

Residual fibrous scar tissues with sufficient volume and biomechanical strength could be shaped into a natural alternative to the patella through soft tissue balancing, and our function-preserving strategy combining semi-constrained TKA with postoperative rehabilitation might realize an effective and safe post-patellectomy recovery.

## 1 Introduction

The patella, a core component supporting knee extension, exerts biomechanical functions by lengthening the quadriceps lever arm, optimizing pull direction, enhancing extension torque, maintaining joint stability, and protecting the femoral trochlea (1, 2). Patellectomy is a traditional surgical procedure for treating comminuted fractures, severe infections, or tumors in the patella (3–5). However, it alters the instantaneous center of knee rotation that requires a 30% increase in the quadriceps strength to support knee extension following the replacement of the resected patella with an iliac bone graft, often leading to long-term, severe, secondary knee osteoarthritis (6).

The demand for total knee arthroplasty (TKA) is surging with the aging population, posing a challenge on a special cohort of individuals with a history of patellectomy (7, 8). After removal of the patella, anatomical changes bring with controversies over the necessity of reconstructing of the bony patella, the way to realign the knee extensor mechanism, and the optimal level of constraint for TKA. Autologous bone grafting for patella reconstruction aids in restoring the mechanical leverage, but carries high risks of complications like graft resorption, nonunion, and fractures (9). In recent years, a non-reconstruction strategy for TKA has been proposed, highlighting the importance of functional preservation of *in situ* scar tissues through meticulous soft tissue balancing (10, 11).

This study retrospectively reported the successful treatment of a case with severe osteoarthritis secondary to patellectomy 40 years earlier, using the “function-preserving strategy” via fibrous scar tissue extension in TKA and a postoperative rehabilitation program. Our experience may provide references for broadening the clinical application of TKA to post-patellectomy osteoarthritis.

## 2 Case presentation

### 2.1 Case details

A 59-year-old male patient was admitted in 2024 with a 10-year history of recurrent left knee pain and limited mobility, which had worsened over the previous 6 months. He was treated with a left patellectomy due to severe multiple traumas 40 years earlier. Post-patellectomy knee function was acceptable in the short-term period, but gradually declined, manifested as limited mobility and pain over the decade earlier. Symptoms worsened within the six months before admission, restricting the maximum walking distance of only 500 meters and the ability to independently squat or climb stairs.

Physical examinations showed visible old surgical scars on the frontal and posterolateral skin of the left knee, presenting with a mild varus deformity. A positive tenderness in the medial knee space was palpated. The left patella was non-palpable, but a firm, non-mobile mass measuring approximately 3.0 cm × 2.0 cm was anterolaterally located on the left knee. Skin temperature around the left knee was normal. The range of motion (ROM) of the left knee was reduced to 10°−90°, and that of the right knee remained normal. Crepitus in the left knee was heard during movement. The left knee was graded with negative results in the lateral stress and drawer tests. No significant abnormalities were found in the 10 cm circumference measurement above the patella. Pathological signs were absent.

CT three-dimensional imaging showed that the space of the left knee joint was significantly narrowed, especially in the medial side (Figures 1a, b). Osteophytes developed extensively along the joint margins. The femoral trochlea had grown into abnormally shallow and flat after a left patellectomy. The full-length radiograph of both lower limbs showed that the hip knee ankle angle was about 173 degrees (Figure 1C).

**Figure 1:**
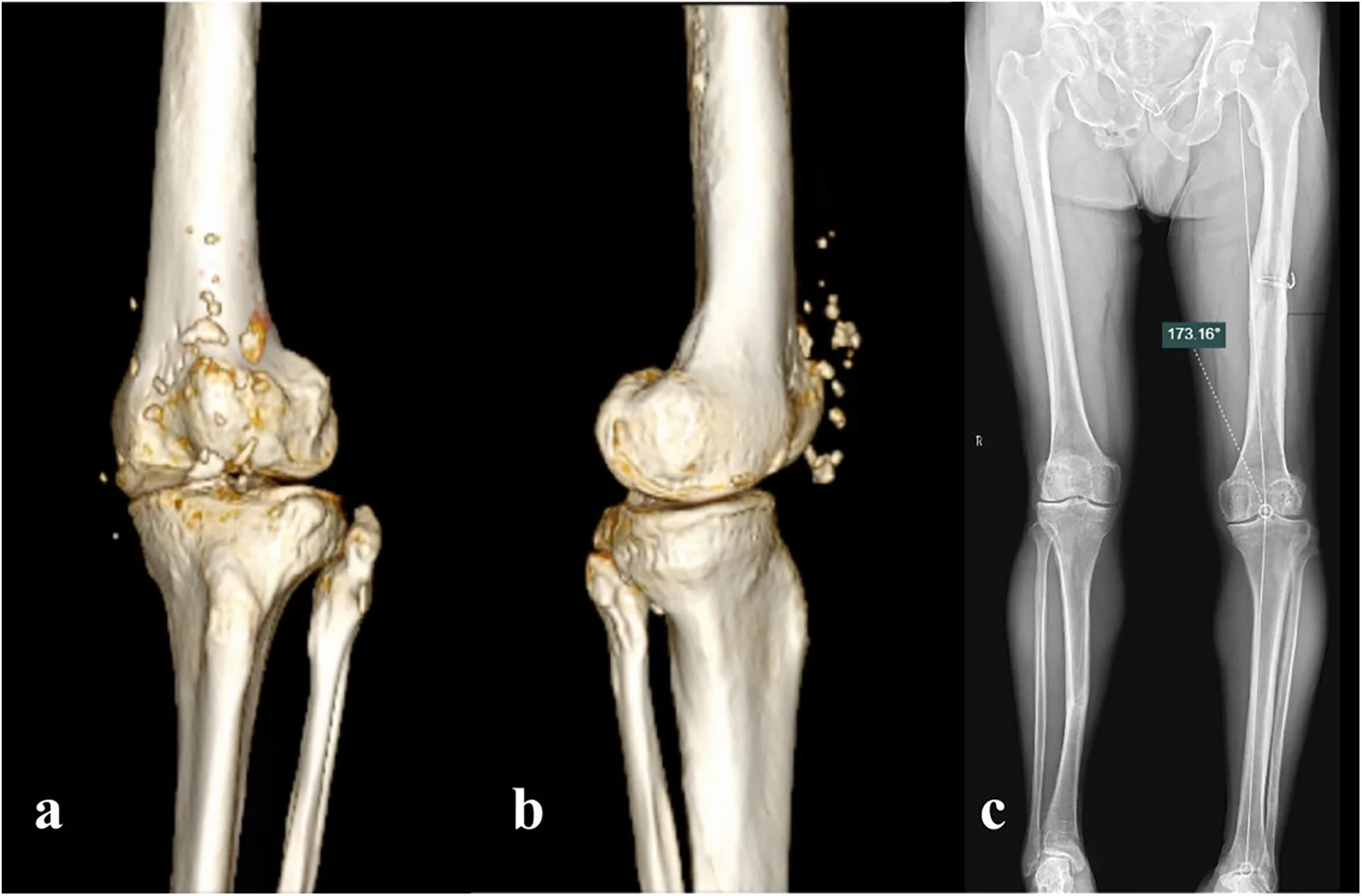
Preoperative 3D CT imaging of knee joint **(a** and **b)**; the preoperative full-length anteroposterior x-ray of lower extremities **(c).**

**Figure 2:**
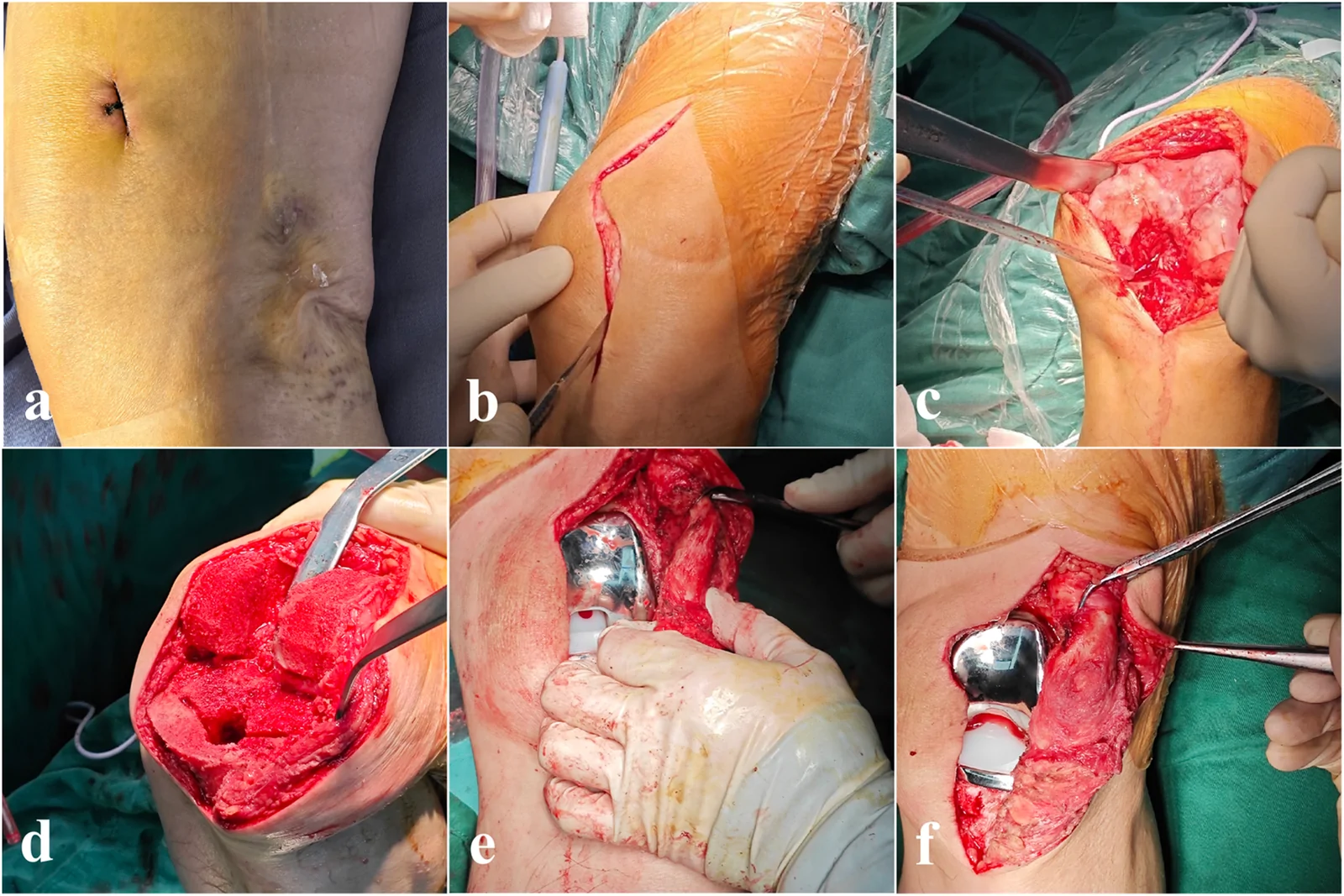
Surgical procedures. **(a)** Clinical photograph showing old surgical scars on the left knee. **(b)** A 15-cm midline incision on the anterior aspect of the left knee. **(c)** Intraoperative exploration reveals the absence of the left patella. **(d)** Routine osteotomy. (e and f) Implantation of a semi-constrained prosthesis, repositioning the fibrous scar tissues to the original site of the patella.

The Ethics Review Board of the Mianyang 404 Hospital approved this study (2026-EC-015). The study was conducted following the tenets of the Declaration of Helsinki (as revised in 2013). The patient provided written informed consent for the inclusion of their data.

### 2.2 Preoperative planning

Soft tissue coverage and integrity for the left knee extensor mechanism were assessed preoperatively. A surgical strategy was tailored based on his conditions in intraoperative exploration. Briefly, fibrous scar tissues with sufficient volume and biomechanical strength would be trimmed and prepared, via soft tissue balancing, for a fibrous pseudo-patella, followed by a semi-constrained TKA (plan A). Without qualified fibrous scar tissues, a cancellous bone block would be harvested from the distal femoral condyle to reconstruct the patella, followed by a fixation with non-absorbable sutures between the quadriceps tendon and patellar tendon (plan B).

### 2.3 Surgical procedures

Prior to the surgery, the patient received general anesthesia, with a tourniquet applied to the upper thigh. A 15-cm midline incision was made on the anterior side of the left knee, followed by the excision of old surgical scar tissues and preparation of an access through the medial femoral muscle space. Intraoperatively, the congested, edematous, and hyperplastic intra-articular synovium was thoroughly debrided. A dense, fibrous, sclerotic mass (4.0 cm × 3.0 cm × 1.5 cm), with a hard, cartilage-like, and non-elastic texture, was located in the original position of the patella, continuing proximally with the quadriceps tendon and distally with the patellar tendon. Based on his conditions, the patient was surgically treated with soft tissue balancing and semi-constrained TKA (plan A), in which the fibrous scar tissues were used as a natural alternative to the patella. Briefly, the mass edges were trimmed with a scalpel into the elliptical shape of a patella, and its inner surface was smoothed to accommodate the prosthetic trochlea. The lateral retinaculum was extensively released until reaching the anterior border of the iliotibial band, thus countering the lateral subluxation of the knee extensor caused by long-standing alignment abnormalities. Medial capsular tightening was performed using an imbrication technique (vest-over-pants type), in which the medial capsule was overlapped and secured with interrupted sutures (#2 non-absorbable suture). After confirming the central position of the fibrous pseudo-patella within the femoral trochlea, the lower limb alignment was corrected via the conventional osteotomy. Considering the loss of anterior restraint and the potential risk of instability after patellectomy, a semi-constrained (constrained condylar knee, CCK-type) prosthesis (Beijing Lidakang Technology Co., Ltd., Tibial Pad RYC402, Tibial Support RYB402, Femoral Condyle RYA202) with long intramedullary stem extensions on both the femoral and tibial components and a varus-valgus constrained polyethylene insert was implanted to enhance tibial plateau stability and limit abnormal anterior translation of the femur relative to the tibia (Figures 3a, b).

**Figure 3:**
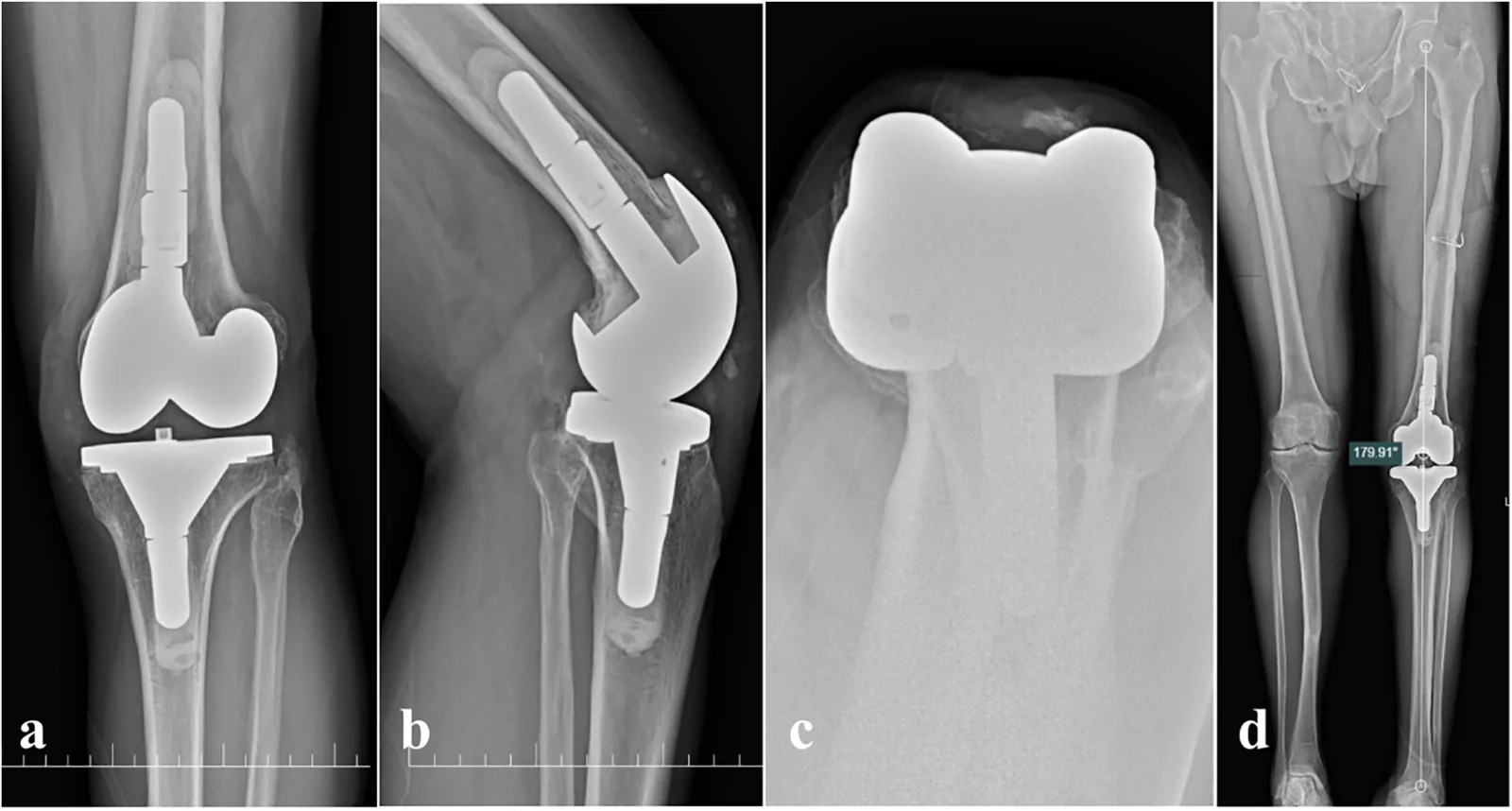
Postoperative anteroposterior **(a)** and lateral radiographs of the left knee **(b) (c)** the postoperative axial skyline radiographs. **(d)** The postoperative full-length standing radiograph of bilateral lower limbs.

After trialing the component placement, the full-range flexion-extension testing validated an active ROM of the left knee. The fibrous pseudo-patella exhibited smooth tracking within the trochlear groove of the femoral component, without any crepitus, impaction, or dislocation. Finally, a permanent prosthesis was implanted. Following the irrigation, hemostasis, and drainage on the surgical site, the incision was closed in layers. During the 110-min surgery, approximately 300 mL of intraoperative blood loss was recorded.

### 2.4 Postoperative rehabilitation

The patient was postoperatively treated with routine antimicrobial, anticoagulant, and analgesic therapies. Aiming to strengthen progressive quadriceps muscle, rehabilitation was initiated immediately on postoperative day 1, lasting for 12 weeks as follows. (1) Postoperative 1–3 days: isometric quadriceps contractions and ankle pump exercises, with 10–15 min per cycle for a total of 3–4 cycles daily; (2) postoperative 4–7 days: straight leg raises and passive-assisted ROM for 2–3 cycles daily; (3) postoperative 2–4 weeks: active-assisted knee flexion/extension and continuous passive motion (CPM) therapy targeting knee flexion at 90°; (4) postoperative 4–8 weeks: intensified active flexion/extension training, progressive weight-bearing walking, and quadriceps resistance training; (5) postoperative 8–12 weeks: functional activity training (e.g., stair climbing/descending, squatting) and gait correction exercises. A combination of closed-chain (e.g., wall squats) and open-chain exercises (e.g., straight leg raises) were performed to restore the active knee extension capacity.

## 3 Results

Significantly, the surgery relieved the pain in the left knee. Immediately after the surgery, the left knee resumed a full passive knee extension with an active extension strength slightly lower, regained functional knee ROM of 25°−120°. The active knee extension was generally restored, and the quadriceps strength was markedly improved at 6 weeks of systematic rehabilitation. At the 3-month follow-up, the patient regained functional knee ROM of 0°−120°, and was able to independently squat, stand up, and walk without walking aid. At the 9-month follow-up, the patient could present a normal gait pattern, and walk 18,000–20,000 steps daily. The functional knee ROM is maintained within the normal range of 0°−120°Meanwhile, the hollow at the front of the left knee disappeared. At 1 year postoperatively, prosthesis positioning was visualized as optimal, and fibrous sclerotic tissues stable on radiographs, without signs of loosening or wear (Figure 3). The hip knee ankle angle has returned to nearly 180 degrees. The Knee Society Score (KSS) of the patient increased from 24 points before operation to 82 points after operation. The patient was highly satisfied with pain relief and functional recovery.

## 4 Discussion

The knee extensor mechanism directly contacts the distal femur following a patellectomy, resulting in a significantly shortened lever arm. Biomechanical data reported a 30% post-patellectomy increase in the contraction force of quadriceps essentially for generating an equivalent knee extension torque. The anatomical anomaly after patellar resection poses three major challenges on individuals to be treated with TKA, including the insufficient knee extension force and delayed extension, the susceptibility to lateral subluxation of the extensor mechanism, and the lower anteroposterior joint stability prone to femoral anterior displacement. Whether to construct a patella or not is also controversial in this special population receiving TKA. Autologous or allograft bone grafting, a traditional procedure to restore mechanical leverage in individuals with absent patellae, brings complications like graft fractures, avascular necrosis, and fixation failure (12, 13). Liu et al. reported a successful reconstruction of resected patella using 3D-printed porous tantalum metal (14). However, this is a complex technique relying on expensive custom prostheses and meticulous intraoperative fixation, hindering its extensive clinical application. In this case report, the patient suffered severe osteoarthritis secondary to patellectomy performed 40 years earlier. We proposed a function-preserving strategy, a novel treatment established in the body's self-compensatory mechanisms, to surgically create a fibrous pseudo-patella through soft tissue balancing prior to TKA. Intraoperative exploration revealed that the *in situ* fibrous scar tissues had grown into a cartilage-like hard mass continuing proximally with the quadriceps tendon and distally with the patellar tendon after 40 years of remodeling, serving as a potential natural alternative to the patella. Intraoperatively, disordered scar tissues were first reshaped into patella-like to accommodate the prosthetic trochlea, followed by trajectory optimization to achieve an optimal alignment within the trochlear groove. The tendency of the extensor mechanism to dislocate was prevented through soft tissue balancing via lateral retinaculum release and medial capsular tightening. After medial capsular tightening, we repeatedly assessed patellar tracking through a full range of flexion, and no eversion or lateral subluxation was observed. Furthermore, postoperative imaging and clinical follow-up confirmed that the pseudo-patella remained stable without eversion. Superior to bony reconstruction, the function-preserving strategy was less invasive and more economically friendly, greatly reducing donor-site injuries and implant-related complications.

Patellectomy disrupts the anterior-posterior stability of the knee joint. Khaimov et al. recommended the selection of TKA prostheses to post-patellectomy patients based on individual intraoperative ligament status (15). The standard posterior-stabilized (PS) prostheses, developed relying on a cam-post mechanism, bring about a risk of post wear under long-term high stress. Therefore, a semi-constrained prosthesis was implanted in this patient, which effectively compensated for patellectomy-induced mild joint instability, thus providing a high safety for early functional rehabilitation (16). In the early postoperative period, the active extension of the left knee was still limited, indicating that the surgical procedures of fibrous scar tissue extension and TKA alone did not immediately restore the functional ability of the knee joint to straighten fully. Postoperatively, the patient was managed by a targeted rehabilitation to strengthen intensive and sustained quadriceps, with an intensity and a duration exceeding those of conventional post-TKA exercises. Postoperative rehabilitation, an essential part in the function-preserving strategy, effectively counteracted mechanical lever disadvantages via compensatory muscle hypertrophy and enhanced neuromuscular control. In our case report, the excellent postoperative functional recovery of the left knee was largely attributed to his strict adherence to the rehabilitation regimen.

Although short-term functional outcomes in this case were satisfactory, long-term risks warrant attention. Without the protection of the patella, the quadriceps tendon is directly exposed to the contact with the prosthetic trochlea, thus bringing a risk of tendon abrasion and rupture. Additionally, the wear resistance of residual fibrous scar tissues that operate as cushions requires a long-term observation. Furthermore, the survival and revision rates of prostheses in post-patellectomy individuals treated with TKA should be analyzed in large-scale, long-term clinical studies (17).

## 5 Conclusion

Overall, a patient with severe osteoarthritis secondary to patellectomy 40 years earlier was successfully treated with the function-preserving strategy via fibrous scar tissue extension in semi-constrained TKA and a postoperative rehabilitation program aiming to strengthen the quadriceps femoris muscle. Individualized intraoperative assessment and decision-making are crucial for the special cohort of post-patellectomy individuals ready to receive TKA. Residual fibrous scar tissues with a tough texture and a moderate volume in intraoperative exploration may serve as a fibrous pseudo-patella to ensure the safe and effective surgical procedures of semi-constrained TKA. Three key factors determine the success of the function-preserving strategy in post-patellectomy individuals surgically treated with TKA, including the selection of a semi-constrained prosthesis to enhance joint stability, a centered trajectory of the extensor mechanism through soft tissue balancing, and an intensive quadriceps rehabilitation postoperatively to compensate for the loss of the lever arm. Overall, the function-preserving strategy simplifies the surgical process of TKA, reduces the risk of complications, and achieves a smooth functional recovery, greatly benefiting post-patellectomy individuals.

## Data Availability

The original contributions presented in the study are included in the article/Supplementary Material, further inquiries can be directed to the corresponding author.
